# Verbal Autopsy as a Tool for Defining Causes of Death in Specific Healthcare Contexts: Study of Applicability through a Traditional Literature Review

**DOI:** 10.3390/ijerph191811749

**Published:** 2022-09-17

**Authors:** Paolo Bailo, Filippo Gibelli, Giovanna Ricci, Ascanio Sirignano

**Affiliations:** Section of Legal Medicine, School of Law, University of Camerino, Piazza Cavour, 19, 62032 Camerino, Italy

**Keywords:** verbal autopsy, cause of death, complementary methodology, pandemic, morbid condition

## Abstract

Autopsy examination, the gold standard for defining causes of death, is often difficult to apply in certain health care settings, especially in developing countries. The COVID-19 pandemic and its associated difficulties in terms of implementing autopsy examinations have made the need for alternative means of determining causes of death even more evident. One of the most interesting alternatives to the conventional autopsy is the verbal autopsy, a tool that originated in Africa and Asia in the 1950s and consists of a structured interview with the deceased’s family members concerning the symptoms manifested by the person and the circumstances of death. In the early 1990s, the first doubts emerged about the validity of verbal autopsies, especially about the real reliability of the cause of death identified through this tool. The objective of the review was to identify studies that had assayed the validity of verbal autopsies through a rigorous comparison of the results that emerged from it with the results of conventional autopsies. When starting from an initial pool of 256 articles, only 2 articles were selected for final review. These are the only two original research articles in which a verbal autopsy validation process was performed by employing the full diagnostic autopsy as the gold standard. The two papers reached opposite conclusions, one suggesting adequate validity of verbal autopsy in defining the cause of death and the other casting serious doubts on the real applicability of this tool. Verbal autopsy undoubtedly has extraordinary potential, especially in the area of health and demographic surveillance, even considering the implementation that could result from the use of artificial intelligence and deep learning. However, at present, there appears to be a lack of solid data to support the robust reliability of this tool in defining causes of death.

## 1. Introduction

Determining the cause of death of an individual turns out to be extremely important in the context of modern society, as it has several implications of both a social and a medical-healthcare nature [1]. The conventional classical method for determining the cause of death is the performance of the autopsy, which, if carried out in a technically adequate manner, sufficiently close to the time of death, and with additional laboratory investigations (mainly toxicological and histopathological), allows the cause of death to be identified with a high degree of accuracy [2,3]. Autopsy also turns out to be frowned upon in modern society by family members as it is seen as disrespectful to the dead person and an obstacle to their burial [4,5]. In Italy, autopsy examinations are ordered by the health and judicial authorities, and it is not possible to object to the same procedure as it is not provided for in the legislation itself [6].

In the COVID-19 pandemic context, the performance of autopsies has been challenged, as in the early stages, they were only authorized in specific centers to avoid the risk of infectious transmission, and later, new centers were created to come to grips with the numerous requests [7]. In addition, the huge number of victims of the pandemic itself actually prevented, due to material and logistical issues, the performance of all the autopsies that would have been necessary [8]. Plural autopsy investigations were postponed, compromising the very best of the autopsy technique due to the intervening degradation processes, and others were never performed with the assignment of a generic cause of death [9]. This situation has caused and is still causing problems of a clinical–epidemiological nature due to the lack of certain data to allow the optimal study of the pandemic phenomenon and monitoring of other morbid conditions [10]. In addition, there have been further problems in the courts for the attribution of medical malpractice in the absence of a certified cause of death, which has not made it possible to identify those responsible or to exonerate the intervening health care providers.

In the search for possible solutions to this problem, we considered verbal autopsy, a pragmatic methodological approach to collecting cause-of-death data in settings where autopsies are not routinely performed, such as in Africa, Asia, and Oceania [11]. Verbal autopsy methodology is based on the collection of anamnestic data by an oral interview with a close relative or caregiver of a person who died within a short time of death: these data include symptoms, signs, and circumstances prior to death [12]. These collected data are analyzed through computer models and by health care personnel with the attribution of the most probable cause of death [13]. The baseline questions were collected within the so-called “Verbal Autopsy Instrument”, which was developed by the WHO in 2012 and last revised in March 2022 [14].

A verbal autopsy has also been employed in the COVID-19-related pandemic context achieving good results [15]. Given the need to perform the anamnestic interview with relatives more methodical before performing an autopsy, and also considering scenarios of undoubted severity (e.g., the pandemic) where autopsies fail to be routinely performed, one possible solution is to import the methodology of verbal autopsy to supplement the autopsy assessment. A key concept that must be emphasized is that, given the profoundly different nature of the two investigations, the verbal autopsy cannot be regarded as a true substitute for the conventional autopsy but, at most, a useful instrument for supplementing the investigation and integrating the autopsy findings.

In order to assess the real reliability of the findings obtained through verbal autopsy, we carried out a review of the current literature to verify whether there are technical prerequisites to imagine that verbal autopsy can be profitably employed to determine the causes of death in specific healthcare contexts.

## 2. The Verbal Autopsy: Birth and Evolution

Verbal autopsy originated in Asia and Africa around the late 1950s, when researchers began investigating population causes of death for purely statistical purposes through interviews with family members of the deceased. This method was first called “Verbal Autopsy” within the Narangwal Project in India in 1956 [16]. In the 1970s, the World Health Organization encouraged the use of this survey modality by people without medical training. Attempts to make this practice more organic and systematized came in the first half of the 1990s when the WHO developed standards on how to conduct a verbal autopsy to investigate maternal deaths. This was followed in 2007 by the development of three more verbal autopsy models (‘death of a child aged under four weeks’, ‘death of a child aged four weeks to 14 years’, and ‘death of a person aged 15 years and above’) [17]. This was accompanied by certification of the cause of death coded according to the Classification of Diseases and Related Health Problems, 10th revision (ICD-10), and a blind verification process, with the cause of death assigned by three different physicians for each case; if at least two out of three physicians agreed, the coding was deemed valid (Physician-Certified VA, PCVA). These models were then updated and refined in 2012, 2014, 2016, and 2022 [18]. Particularly important was the 2016 intervention by which the WHO revised VA standards to make them fully compatible with computer-coded verbal autopsy (CCVA) algorithms available on public domains [17]. A verbal autopsy has a very useful use today in developing countries, where it plays an essential role in health and population surveillance. For example, it has been successfully used in Senegal to test maternal mortality, infant mortality, and malaria mortality in rural settings [19,20,21]

Future prospects see effective integration of current verbal autopsy systems with artificial intelligence and deep learning systems [22]. A verbal autopsy also seems to be able to establish itself as the main tool for determining the cause of death in those countries where for religious reasons, a conventional autopsy is seen as an impermissible form of outrage against the deceased. In practice, the verbal autopsy is divided into two stages. The first stage consists of gathering the necessary information (medical history, signs, and symptoms presented near death, circumstances of death) through interviews conducted with the family members and caregivers of the deceased. The second stage consists of interpreting the data in order to identify the cause of death. This interpretive process can be performed by humans, in this case, physicians (the so-called Physician-Certified VA), or by computer systems (Computerised Coding of VA), such as InterVA and SmartVA.

## 3. Aims and Objectives

One of the major weaknesses of the verbal autopsy is the strong doubts about its real ability to identify the actual cause of death. The objective of this review is to identify scientific papers from the past 20 years that have investigated the reliability of verbal autopsy findings through a comparison with the findings of the conventional autopsy examination. A literature review was conducted, including papers published between 2001 and 2022, according to a systematic search strategy.

## 4. Searching Strategy

The research was carried out on the scientific literature between January 2001 and May 2022 in the online databases of PubMed, Scopus, and Web of Science (WoS). The Pubmed database search was performed by combining the Mesh terms “verbal autopsy” [All Fields] and “validation” [All Fields] with the “AND” Boolean operator. We conducted the search on Scopus by entering the following search term: “verbal autopsy”. Then we performed a new search by inserting the following terms: “Validation”, “Validation process”, “Validation study”, and combined them with the operator “OR”. We set “title-abstract-keywords” as a field tag. Finally, we combined the results of the two searches with the AND operator. The search on Web of Science was conducted similarly to that on Scopus, the only difference being that we entered “title-abstract” as the tag field.

We performed a preliminary skimming independently: each author read the abstracts of the articles found and identified those they considered useful for the review. At the end of the preliminary evaluation procedure, the authors discussed the various articles, debating the suitability of the individual papers. At the end of the selection phase, the authors read all the articles in order to collect the data for the review.

## 5. Selection Criteria

The research initially provided 256 results. Specifically, 86 papers were found in PubMed, 109 in Scopus, and 61 in Web of Science. The types of study objects of interest were the following: original articles, review articles, book chapters, conference papers, editorial materials, proceeding papers, and meeting abstracts. We made an initial pre-selection by removing duplicate papers (*n* = 150), articles not written in English (*n* = 0), and articles for which the full text was not available (*n* = 1). We then read the abstracts of the remaining 106 articles in order to identify papers suitable for reading the full text. At this stage, we decided to include only those articles that specifically addressed validation of the verbal autopsy by comparison with full autopsy findings. After reading the abstracts, we excluded 104 articles as not relevant to the purposes of the review. Specifically, 51 papers were related to areas completely unrelated to the topic of interest (e.g., report on specific illness; collection of verbal autopsy data), 23 papers discussed the validation of verbal autopsy using medical records, and 31 papers discussed the validation process of algorithm versus the clinician interpretation of verbal autopsy data. We then proceeded to read the full text of the remaining two papers, which were both included in the final review.

## 6. Quality Evaluation

SANRA (Scale for the Assessment of Narrative Review Articles) [23] was employed for quality checks of selected studies. The overall quality was determined as poor (score 0–6), moderate (7–9), and excellent (10–12). The two articles were found to be of excellent quality. The results of SANRA are reported in Table 1.

**Figure 1:**
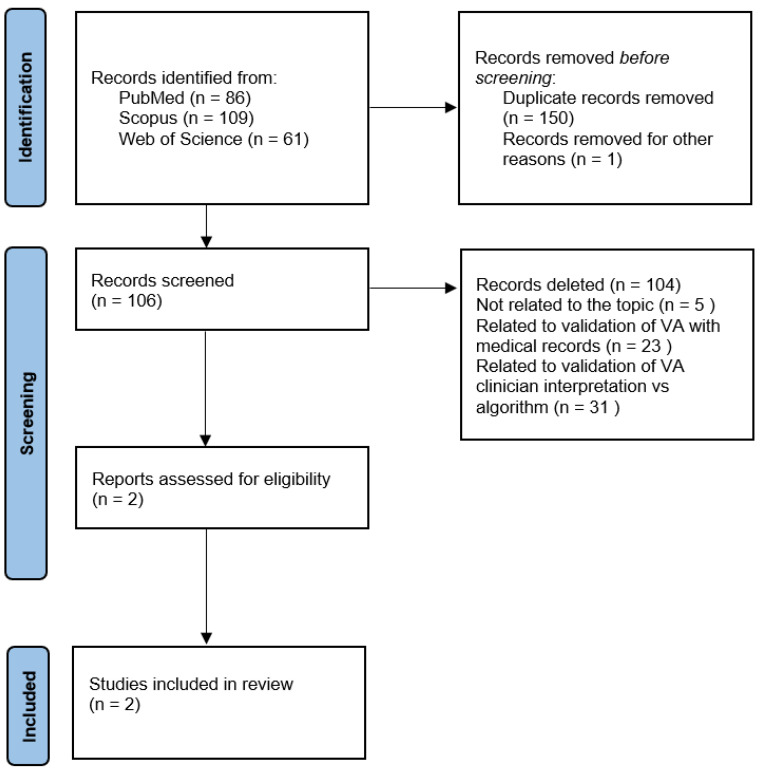
Review search strategy.

Figure 1 illustrates the diagram of the article selection process.

## 7. Summary of Article Pool

The search identified two articles suitable for inclusion in the review. Of the two articles, one was published in 2021 and one in 2022. The socio-environmental and cultural background of the articles is Mozambique and Brazil. Regarding the types of articles, they are original research articles in which the authors perform a validation process of the verbal autopsy by using as the gold standard the conventional diagnostic autopsy. Table 2 illustrates the main characteristics of the reviewed articles.

## 8. Results

The research showed that there are almost no validation studies of the verbal autopsy performed with the real gold standard, which should be the conventional diagnostic autopsy. Most of the articles found performed validation using medical records considering this technique as the gold standard and addressed the problem of validation of the verbal autopsy on the interpretation of the data itself: showing differences depending on the interpretation performed by algorithms vs. physicians. Only two articles were found that address the problem of validation by comparing the result of the verbal autopsy with that of a conventional diagnostic autopsy.

The first [24] is a study performed on 316 deceased patients in Mozambique. The VA forms were compiled through the information provided by the attending physician from the clinical record of the deceased individual and from the obstetric record in cases of perinatal deaths. The patients consisted of a heterogeneous group taking into consideration both childhood and adulthood and deaths in the context of pregnancy. Menéndez et al. show overall that the verbal autopsy tool turns out to be inaccurate and insufficient in the wake of the dissimilarities of the results obtained, showing weakness, especially regarding the identification of specific infectious diseases, neoplasms, and some issues related to pregnancy and the newborn. Ultimately, the authors suggested extending autopsy investigations and improving verbal autopsy techniques.

The second study [25] was performed in Brazil on a sample of 3139 adult deceased patients, 2060 from São Paulo and 1079 from Recife. Causes of death were processed by collecting information from family members and using the SmartVA-Analyze program, implemented with PCVA only for the 2060 subjects in São Paulo. All subjects underwent conventional autopsy. The cause-specific mortality fractions estimated using SmartVA turned out to be broadly similar to autopsy for cardiovascular diseases, cancers, infections, and chronic respiratory diseases. Compared to the autopsy, the SmartVA estimates were lower for the other non-communicable diseases and higher for diabetes.

## 9. Discussion

The diagnosis of death ascertained by an autopsy performed by a pathologist appears to be the gold standard for determining the cause of death of an individual [2]. Despite the undoubted usefulness of autopsy, there is a decline in the number of autopsies performed, both due to logistical factors [26] and a growing distrust of autopsy practice [4,5]. Moreover, in particular situations (e.g., the COVID-19 pandemic), the possibility of performing autopsy investigations appears to be even more diminished. Therefore, we investigated the possibility of implementing/revising the classical autopsy assessment with the verbal autopsy tool, which is mainly used in African, Asian, and Oceania countries [12]. In order to enable good reliability of this tool, it is necessary to verify, by means of validation techniques, how well the verbal autopsy gives overlapping results with respect to a full diagnostic autopsy. In order to do this, a comprehensive review of the available scientific literature was performed, but only two papers were found that validate verbal autopsy with actual autopsy data.

The two studies come to opposite conclusions: Hart et al. [25] judge verbal autopsy to be an effective tool and show encouraging results of the validation process, while Menéndez et al. [24] turn out to be skeptical about a verbal autopsy, showing all the dissimilarities with the causes of death of full diagnostic autopsy. It is quite evident that the field of verbal autopsies has developed in countries where difficult economic conditions have prevented checking the causes of death using the gold standard of full autopsy [11]. Thus, efforts have been made to still allow the determination of causes of death through simple anamnestic collections obtained from the relatives of the deceased. The effort of the scientific community has mainly focused on validating the technique by relying on medical records and reading/interpreting the data itself, which can be performed by physicians or through algorithms [27].

In the absence of a concrete basis for validation of verbal autopsy by comparison with full diagnostic autopsies, extending this tool to other geographical areas appears to be impractical. Therefore, more validation studies are desirable so that the verbal autopsy can be refined and rendered with results almost superimposable to the full autopsy assessment. In addition, in the study by Hart et al. [25], it is shown how the pathologist benefits from accessing the raw anamnestic data collected during the verbal autopsy interview and is better able to target the cause of death. Based on this observation, it will be possible to use verbal autopsy as a complementary method to the full diagnostic autopsy, with synergistic input from both tools.

## 10. Conclusions

A verbal autopsy is a tool for determining causes of death that is destined to play an increasingly central role, both because of advances in its implementation and because of the likely spread of future pandemics (of which COVID-19 was only the first). Despite the fact that conventional autopsy remains a centrally important tool even in pandemic settings (as it was in the context of COVID-19), being able to rely on an alternative but equally accurate mode of ascertaining causes of death could be of extraordinary importance.

The advantages offered by this tool are numerous and unfold on many levels: cost savings, simplicity of execution, overcoming ethical–religious issues, and the possibility of carrying out a thorough investigation aimed at identifying the cause of death when conventional autopsy examination is not possible.

However, despite being an extremely promising investigation modality, at present verbal autopsy does not yet seem ready to be used instead of a conventional autopsy. More validation studies performed with comparison not only with medical records but also with autopsy findings are needed. After this evaluation process, it will be a very interesting and promising tool to supplement/vicariate conventional autopsy even in developed country settings. However, there is no doubt that in settings where conventional autopsy examination is not feasible, a verbal autopsy is the best possible alternative.

It should also be noted, in the final analysis, how verbal autopsy has the potential to become an extremely useful tool in the near future not only in the context of accidental or natural deaths but also in the context of forensic investigations, thus, clarifying cases of violent deaths resulting from suicide or homicide. This is especially true in cases of suicide, where a thorough psychological investigation conducted on family members and friends (so-called “psychological autopsy”) can clarify the circumstances of the death of a self-suppressive nature, especially in individuals with mental illness [28,29,30].

## Figures and Tables

**Table 1:** SANRA Score for quality assessment of selected studies for the review.

Reference and Year of Publication	Justification of the Article’s Importance for the Readership	Statement of Concrete Aims or Formulation of Questions	Description of the Literature Search	Referencing	Scientific Reasoning	Appropriate Presentation of Data	Total Score
Menéndez et al. [24] 2021	2	2	1	2	2	2	11
Hart et al. [25] 2022	2	2	1	2	2	2	11

**Table 2:** Summary of the content of the 2 articles included in the review.

Reference and Year of Publication	Socio-Environmental Context	Type of Article	Title	Number and Type of Cases	Overall Results
Menéndez et al. [24] 2021	Mozambique	Research Article	Limitations to current methods to estimate cause of death: a validation study of a verbal autopsy model	316 patients, from stillbirth to adults	Poor validation of VA VA less sensitivity for infectious diseases, neoplasia and pregnancy/newborns pathologies
Hart et al. [25] 2022	Brazil	Research Article	Validation of physician certified verbal autopsy using conventional autopsy: a large study of adult non-external causes of death in a metropolitan area in Brazil	3139 patients, above 18 years (smart VA and PCVA for the 2060 deaths in São Paulo and only smart VA for the 1079 deaths in Recife)	Good validation of VA Opportunity to share anamnestic VA data to pathologists

## Data Availability

Not applicable.
